# AI as a Medical Device Adverse Event Reporting in Regulatory Databases: Protocol for a Systematic Review

**DOI:** 10.2196/48156

**Published:** 2024-07-11

**Authors:** Aditya U Kale, Riya Dattani, Ashley Tabansi, Henry David Jeffry Hogg, Russell Pearson, Ben Glocker, Su Golder, Justin Waring, Xiaoxuan Liu, David J Moore, Alastair K Denniston

**Affiliations:** 1 Institute of Inflammation and Ageing College of Medical and Dental Sciences University of Birmingham Birmingham United Kingdom; 2 University Hospitals Birmingham NHS Foundation Trust Birmingham United Kingdom; 3 NIHR Birmingham Biomedical Research Centre Birmingham United Kingdom; 4 NIHR Incubator for AI and Digital Health Research Birmingham United Kingdom; 5 Springer Nature Group London United Kingdom; 6 School of Chemistry, Food and Pharmacy University of Reading Reading United Kingdom; 7 Population Health Science Institute Faculty of Medical Science Newcastle University Newcastle United Kingdom; 8 Medicines and Healthcare Products Regulatory Agency London United Kingdom; 9 Kheiron Medical Technologies London United Kingdom; 10 Department of Computing Imperial College London London United Kingdom; 11 Department of Health Sciences University of York York United Kingdom; 12 Health Services Management Centre University of Birmingham Birmingham United Kingdom; 13 Institute of Applied Health Research University of Birmingham Birmingham United Kingdom

**Keywords:** adverse event, artificial intelligence, regulatory science, regulatory database, safety issue, feedback, health care product, artificial intelligence health technology, reporting system, safety, medical devices, safety monitoring, risks, descriptive analysis

## Abstract

**Background:**

The reporting of adverse events (AEs) relating to medical devices is a long-standing area of concern, with suboptimal reporting due to a range of factors including a failure to recognize the association of AEs with medical devices, lack of knowledge of how to report AEs, and a general culture of nonreporting. The introduction of artificial intelligence as a medical device (AIaMD) requires a robust safety monitoring environment that recognizes both generic risks of a medical device and some of the increasingly recognized risks of AIaMD (such as algorithmic bias). There is an urgent need to understand the limitations of current AE reporting systems and explore potential mechanisms for how AEs could be detected, attributed, and reported with a view to improving the early detection of safety signals.

**Objective:**

The systematic review outlined in this protocol aims to yield insights into the frequency and severity of AEs while characterizing the events using existing regulatory guidance.

**Methods:**

Publicly accessible AE databases will be searched to identify AE reports for AIaMD. Scoping searches have identified 3 regulatory territories for which public access to AE reports is provided: the United States, the United Kingdom, and Australia. AEs will be included for analysis if an artificial intelligence (AI) medical device is involved. Software as a medical device without AI is not within the scope of this review. Data extraction will be conducted using a data extraction tool designed for this review and will be done independently by AUK and a second reviewer. Descriptive analysis will be conducted to identify the types of AEs being reported, and their frequency, for different types of AIaMD. AEs will be analyzed and characterized according to existing regulatory guidance.

**Results:**

Scoping searches are being conducted with screening to begin in April 2024. Data extraction and synthesis will commence in May 2024, with planned completion by August 2024. The review will highlight the types of AEs being reported for different types of AI medical devices and where the gaps are. It is anticipated that there will be particularly low rates of reporting for indirect harms associated with AIaMD.

**Conclusions:**

To our knowledge, this will be the first systematic review of 3 different regulatory sources reporting AEs associated with AIaMD. The review will focus on real-world evidence, which brings certain limitations, compounded by the opacity of regulatory databases generally. The review will outline the characteristics and frequency of AEs reported for AIaMD and help regulators and policy makers to continue developing robust safety monitoring processes.

**International Registered Report Identifier (IRRID):**

PRR1-10.2196/48156

## Introduction

### Background

Patient safety has been defined as one of the 6 key indicators of health care quality by the Institute of Medicine [1]. In a landmark report published in 2001, the Institute of Medicine identified that approximately 100,000 adverse events (AEs) resulted in patient death each year in the United States, with sources suggesting that medical error is third on the list of leading causes of death [2,3]. In the UK’s National Health Service, it is estimated that 8%-12% of hospital admissions may involve an AE resulting in harm to the patient [4,5].

Whereas administration errors and AEs have been extensively explored in the context of medication, there has been much less attention given to similar issues in the context of medical devices [6]. Suboptimal reporting exists due to a range of factors such as failure to recognize the association of AEs with medical devices, lack of knowledge of how to report AEs, as well as a common culture of not reporting events [7]. Evidence shows that a significant proportion of AEs in hospitals (including drug and device AEs) are indeed preventable and that transparency of AE reporting to generate insights into device safety issues is essential [8-10].

Artificial intelligence as a medical device (AIaMD) is an important new tool for improving health care most notably in the areas of diagnosis, screening, prognosis, and clinical decision support systems. There is however an increasingly recognized performance gap between the initial proof-of-concept studies and what may be observed in the more challenging deployment in the real world where there is a greater diversity of setting and population and much less control of external factors [11-13]. An important factor in the wider adoption of AIaMD will be to have adequate systems of safety signal detection, attribution, and reporting that are transparent and can be trusted by health systems and the patients and the public they serve. One key component of existing safety systems is AE reporting. An AE is defined as “An unfavourable outcome that occurs during or after the use of a drug or other intervention but is not necessarily caused by it” [14,15]. It is important to note that definitions of AEs are often centered around the use of medicines rather than medical devices. For the purposes of this review, relevant regulatory guidance such as that from the International Medical Device Regulators Forum (IMDRF) [16] will be used to guide the classification of AEs.

AE reports can be submitted by different stakeholders including device manufacturers, clinical staff or patients, and members of the public. The AE reporting processes vary by country; however, in general, each regulatory body has an AE or safety notice database, of which some are publicly available and searchable. There are currently no specific AIaMD AE reporting processes; and therefore, existing processes for medical devices are currently used.

Guidance from the Medicines and Healthcare Products Regulatory Agency (MHRA) was recently released, aiming to aid manufacturers in understanding what events should be reported [17]. There still remains an urgent need to develop robust safety monitoring processes to ensure that patients, professionals, health systems, and regulators can have confidence that AIaMDs are safe for patients. AE reporting is an important component of safety monitoring and aims to ensure that any AIaMD that is found to be unsafe after reaching the market is swiftly detected and either corrected or removed.

### Purpose

AE reporting is a cornerstone of safety monitoring in medical devices and other health care products and will be a key component of the safe introduction of AIaMD. There are concerns, however, that current AE reporting systems are inadequate with many AEs remaining hidden. Even when AEs are reported, the details are not publicly available, leading to a lack of transparency, and reducing the opportunity for these and other health systems to learn and act early. Even for regulatory territories with stronger AE infrastructure (openly accessible dedicated AE databases), it is unclear how well AEs are reported and recorded within those databases. The systematic review described in this protocol aims to identify reported AEs and data relating to frequency and severity for different types of AIaMD.

The secondary aims of the review described in this protocol are (1) to compare the number of AEs reported for different AIaMD risk classes, (2) to compare AE reporting across 3 jurisdictions with publicly available AE databases, and (3) to identify the level of clinical evidence available for devices with reported AEs.

## Methods

### Reporting of Protocol and Systematic Review

The reporting of this protocol follows the guidelines of PRISMA-P (Preferred Reporting Items for Systematic Review and Meta-Analysis Protocols) [18]. The PRISMA-P checklist is included in Multimedia Appendix 1. Reporting of the subsequent review will adhere to the PRISMA (Preferred Reporting Items for Systematic Review and Meta-Analysis) reporting guideline for systematic reviews [19] and the PRISMA-AI guideline (if available as this is still in development) [20].

### Systematic Review Registration

This systematic review has been registered in the Open Science Framework.

### Information Sources

The search strategy for this review has been designed to identify reported AIaMD AEs. We have identified searchable AE databases through scoping searches across three different jurisdictions: (1) the Manufacturer and User Facility Device Experience (MAUDE)—available from the US Food and Drug Administration (FDA), United States [21]—contains reported AEs associated with FDA-approved medical devices. AEs can be reported by mandatory reporters (manufacturers, importers, and device user facilities) and voluntary reporters (eg, health professionals, patients, and consumers) [21]; (2) The Database of Adverse Event Notifications (DAEN)—available from the Therapeutic Goods Administration (TGA), Australia [22]—similar to MAUDE, contains AE data for medicines and medical devices approved for use by the TGA; and (3) Field Safety Notice (FSNs) website—available from the MHRA, United Kingdom [23]—describes corrective actions taken by device manufacturers in response to an identified safety issue. These notices are not reported AEs; however, they can contain relevant device safety data that can be extracted. FSNs may be disclosed by manufacturers in response to AEs.

Scoping searches have demonstrated that there are relatively fewer information sources for medical device AEs compared to AEs associated with medicines. The 3 databases mentioned earlier have been identified as being searchable to yield details regarding safety events. In addition to the AE or FSN databases, each of the 3 regulatory bodies outlined earlier will be searched to identify any product recalls for AIaMDs.

### Search Strategy

We have undertaken an initial feasibility assessment of searching AE databases. Given that each database is unique to its own regulatory body, different search strategies are required. Search strategies cannot be transferred between AE databases due to inconsistencies in terminology, device names, and search capabilities. It is important to note that the common thread in the search strategies is searching for AIaMDs for which AEs have been reported. The following search strategies are specific to each database listed:

- MAUDE (provided by the FDA)
  This database will be searched using the openFDA application programming interface (API). The openFDA was set up by the first Chief Health Information Officer in March 2013 and enables public access to relevant data including drug and medical device AEs.
  The API can be searched to request several data points. This approach has been used in previous studies to identify AEs for both drugs and devices [24,25].
  The database will be searched to find AEs reported for artificial intelligence (AI) or machine learning (ML)–enabled devices listed on the FDA website [26].
- DAEN (provided by the TGA)
  The DAEN website allows for searches of medical device AEs. Due to a lack of AI- or ML-specific search terminology, it is not possible to search the database using these terms. However, the word “software” is indexed and will be used to search for relevant events. Events specific to AIaMDs will be identified through manual screening.
- FSNs website (provided by the MHRA)
  The MHRA FSN website has an in-built search function, and the database will be searched using a similar approach to the search of DAEN. As mentioned earlier, following the identification of software-related FSNs, manual screening will be undertaken to identify AIaMD-related FSNs.

**Figure 1:**
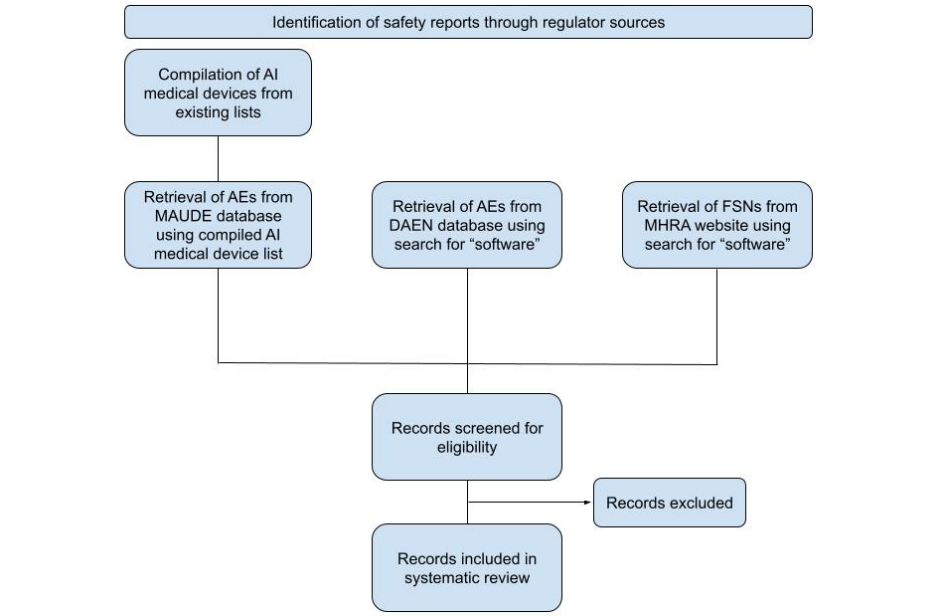
PRISMA flowchart to be populated during the systematic review process. AE: adverse event; AI: artificial intelligence; DAEN: Database of Adverse Event Notifications; FSN: Field Safety Notice; MAUDE: Manufacturer and User Facility Device Experience; MHRA: Medicines and Healthcare Products Regulatory Agency; PRISMA: Preferred Reporting Items for Systematic Review and Meta-Analysis.

For devices where events are identified in one of the 3 information sources, web searches will be used to identify the device manufacturer’s website. Where available, details for clinical evaluations will be identified from the device manufacturer’s website. Data will be extracted for analysis including the type of study, sample size, reported demographics, and whether or not any AEs were identified and reported. Further information regarding the extracted data is listed below, and a PRISMA flow diagram is shown in Figure 1 to be populated during the review process.

### Selection Criteria

#### Intervention

All regulatory-approved AIaMDs will be included in this systematic review. AUK and a second reviewer will independently screen identified AE reports to ensure that the medical device in question is an AI medical device.

#### Types of Documentation

The following documentation will be reviewed to ensure appropriate selection of medical devices:

- MAUDE (provided by the FDA)
  The full AIaMD AE or incident report will be downloaded and cross-referenced with the FDA summary document. The summary documents include details regarding evidence submitted for approval and the date of regulatory approval. This documentation often includes the intended use and type of the AIaMD.
- DAEN (provided by TGA)
  The full AE report will be downloaded and assessed to ensure that the medical device in question is AIaMD. This will be cross-referenced with web-based sources where required, such as the device manufacturer’s website.
- FSNs (provided by MHRA)
  The full FSN will be downloaded and assessed to ensure the medical device is AIaMD. As with the Australian database (DAEN), devices will be cross-referenced with web-based sources as required.

### Selection Process

Once AIaMDs are identified through searches, the AE reports or FSNs will be screened to ensure that the medical device is AI- or ML-enabled. Publicly available regulatory documents and web-based sources will be used to screen devices. Where required, manufacturers will be contacted for additional details. This will be undertaken independently by AUK and a second reviewer. The main inclusion criterion is that the AE report concerns an AIaMD. The exclusion criteria are AE reports for AI health technologies that are not medical devices and AE reports for software as a medical device with no use of AI.

### Risk of Bias Assessment and Confidence in Cumulative Evidence

This systematic review focuses on real-world data reported in regulatory databases. There are no formal risk-of-bias assessment tools for these data; however, the review will include a measure of the completeness of data reported in AE reports. There will be no formal evaluation of confidence in cumulative evidence, as the items included in this review are not research papers.

### Data Extraction

The data extraction process will involve 3 stages. The first stage relates to the MAUDE database (FDA). The openFDA API will be used to extract all AIaMD-related reports into a local database. The data will then be tabulated and displayed in Excel (Microsoft Corp). The second stage relates to the DAEN (TGA) and FSN (MHRA) information sources. For these, the data extraction process will be undertaken using a standardized data extraction form. Data will be entered into the extraction form using Excel. AUK and a second reviewer will extract these data independently using the agreed template, and any disagreements will be escalated to a third arbitration reviewer (AKD or XL). Finally, regulatory data will also be extracted where available for AIaMDs with reported AEs (such as device classification and route to approval). For AIaMDs with reported AEs, published clinical evaluation studies will also be identified where available.

The following data will be extracted based on documentation available from the FDA, the IMDRF, and published literature [27,28]. Data points directly relating to safety reports are type of report, date of event, date of report, patient demographics, patient outcome (death, injury, or malfunction without patient harm), description of the event (free text entry regarding the incident), device brand name (brand name of AI medical device involved in the AE report), manufacturer name (name of the manufacturer for the AI medical device in question), unique device identifier if available, operator of device, reporter occupation, response to AE from the manufacturer where available (details regarding any investigation), latency of response from the manufacturer (time from AE report to manufacturer response), and recall data if available. Further data regarding the AIaMD will be identified from clinical evaluation data submitted to the respective regulatory body (Textbox 1).

**Data points relating to the medical devices in question.**

- Approval number (if Food and Drug Administration approved): Unique approval number assigned to devices acquiring regulatory approval.
- Approval pathway: The name of the regulatory pathway through which artificial intelligence as a medical device (AIaMD) was approved. For example, for the Food and Drug Administration, this will be 510k, de novo or premarket approval.
- Manufacturer origin country: The country of origin of the manufacturer.
- Medical specialty: The medical discipline which the device is intended for.
- Type of artificial intelligence: The type of artificial intelligence system, for example, deep learning.
- Autonomy level of AIaMD: Autonomy level will be graded based on the intended use of the device.
- Intended use of AIaMD: Intended purpose of the device and associated details such as the intended population and setting.
- Risk classification: The risk class of the AIaMD as approved by the regulator.
- Risk level: The risk level of the AIaMD determined according to International Medical Device Regulators Forum guidance.
- Validation sample size: The sample size of the validation cohort if available.
- Retrospective or prospective validation: The type of validation study that was conducted prior to regulatory approval.
- Reported demographics in validation: Any reported patient demographics reported in the validation study.
- Adverse events reported in clinical validation study: Whether or not any adverse events were reported within the validation study prior to regulatory approval.

### Data Synthesis

A descriptive analysis of extracted data will be undertaken. Analysis of the variables described earlier will include comparison across (1) types of intended use (and risk level), (2) specialty areas, and (3) regulatory territories (the United Kingdom or the United States or Australia). The comparison aims to identify whether there are certain types of AIaMD intended use (and risk level), specialty, or regulatory territory within which AEs are more commonly reported. The types of intended use for AIaMDs will be grouped into 2 broad categories based on clinical tasks. These will include (1) diagnostic and prognostic AIaMD and (2) therapeutic AIaMD. Detected AEs and FSNs will be characterized according to AE terminology available from the IMDRF [29].

Once AEs and FSNs have been characterized using AE terminology, rates of each type of AE will be quantified and compared between the groups described earlier. The following further analyses will also be considered:

- The number of AEs reported per year to identify trends in reporting
  Rates of AEs reported for AIaMDs across the 3 jurisdictions mentioned will be calculated. This will be calculated for each group but also across groups to quantify trends in AE reporting.
- Completeness of AE reports
  AE reports often vary in their completeness. For example, some reports may not contain who reported the event or what the final patient outcome was. Completeness of AE reports will be assessed against reference criteria, developed using existing guidance and AE reporting forms. The IMDRF (which has membership of several international regulators) provides guidance relating to AE reporting. This assists in the standardization of AE reports across the globe. Therefore, it is likely that the findings generated will apply to AE reporting both within and outside the 3 jurisdictions included in this study.
- AE reporting party
  The source of the AE will be compared across groups to understand how reporting characteristics vary. For example, reporting by manufacturers versus clinical staff versus patients.
- Availability of clinical evaluation data for medical devices with reported AEs
  Where AEs are reported for AIaMD and clinical evaluation data are available (from regulatory or manufacturer websites), further analysis will be conducted. The types of AEs that are reported across different clinical studies will be identified and compared with AEs detected in AE databases.

The analysis aims to generate insights into the frequency of reporting of AIaMD-associated AEs but also the characteristics of the AEs in order to inform real-world safety monitoring practices for AIaMD. Additionally, this systematic review aims to generate insights into how AE reporting may vary by region and regulatory authority. By understanding what types of AEs have been reported, systems to detect relevant patient harms can be implemented in real-world AI safety monitoring strategies.

## Results

Scoping searches are being conducted with screening to begin in April 2024. Data extraction and synthesis will commence in May 2024, with planned completion by August 2024. The review will highlight the types of AEs being reported for different types of AI medical devices and where the gaps are. It is anticipated that there will be particularly low rates of reporting for indirect harms associated with AIaMD. The results are expected to be submitted for publication in quarter 4 of 2024.

## Discussion

### Overview

The systematic review outlined in this protocol aims to identify and characterize AEs arising from AIaMDs as estimated through AE reporting systems. The review will highlight the types of AEs being reported for different AI medical devices and more importantly where the gaps are. It is anticipated that reporting will be poor overall with particularly low rates of reporting for indirect harms associated with AIaMD. AEs are often poorly reported in the literature and are usually not the primary outcome of clinical research studies [30]. Once a medical device has gained regulatory approval and is in active use, AE reporting is an essential component of postmarket safety monitoring. There is variable reporting of AEs for medicines and medical devices, and reporting can often be sporadic for the latter [6,31]. AIaMD has gained much interest recently, with many new commercially available AIaMDs ready for deployment. However, there remains a lack of consensus on how to ensure robust safety monitoring post deployment. One key aspect is AE reporting for AIaMDs.

### Anticipated Principal Findings

There is minimal awareness around what consists of an AE for AIaMDs and how these AEs should be reported. This systematic review protocol describes the methodologies for searching AE databases and highlights relevant information sources. The systematic review will outline the methodology for searching AE databases and highlight all reported AEs for AIaMD. The review will provide insights into the types of AEs that may occur for AIaMDs, the completeness of reporting, and the frequency of AE reports. Given the often-variable AE reporting for many other types of medical devices, it is likely that AE reporting for AIaMDs will be equally variable if not even poorer. The data points listed in the previous section will allow the analysis of frequency and types of AEs being reported, in addition to the types of AI medical devices for which AEs are more commonly identified. Furthermore, for devices where validation data are available from regulators, the analysis will highlight the potential correlation between the level of evidence submitted for regulatory approval and the likelihood of AEs being reported.

### Limitations

There are several limitations that need to be considered. First, the review described in this protocol focuses on real-world evidence in the form of AEs, which have been reported through postmarket surveillance pathways. This means that there will be variations in the quality of reports. As part of this review, the completeness of the report will be assessed; however, there will not be a formal risk of bias assessment. Second, the scope of this review is focused on AIaMD. There will be numerous AEs reported for software as a medical device without AI. Many of the AEs related to this group of devices could provide relevant insights for AI medical devices, and there might be value in a review for these devices in the future. Finally, this study only examines publicly available data, as access to additional data is reserved for medical device regulators only. It is anticipated that the review will highlight the need for transparent databases, providing a foundation for further work with regulators to reduce the opacity of such data sources.

### Conclusions

This review of AE databases comes at a key milestone in AIaMD regulation reform and aims to inform regulators and policy makers of the current state of AE reporting. The review will provide information such as the devices and types of algorithms for which AEs are most commonly reported, whether any use cases or specialties are frequently involved, and potentially the level of clinical validation associated with frequent AE reports if this is available. A companion systematic review is being conducted by our group investigating AE reporting in randomized controlled trials of AI medical devices. The companion review aims to assess the types of AEs being reported in randomized controlled trials but also how algorithmic performance error analysis is conducted. Together, these systematic reviews will identify the current status of AE reporting across both clinical trials and real-world deployment phases. This work aims to generate insights into how AIaMD safety signals could be monitored going forward while informing the decision-making of multiple stakeholders including manufacturers, AIaMD deployment teams, regulators, and policy makers.

## Appendix

Multimedia Appendix 1 PRISMA-P (Preferred Reporting Items for Systematic Review and Meta-Analysis Protocols) checklist.

## Abbreviations

AE: adverse event
AI: artificial intelligence
AIaMD: artificial intelligence as a medical device
API: application programming interface
DAEN: Database of Adverse Event Notifications
FDA: Food and Drug Administration
FSN: Field Safety Notice
IMDRF: International Medical Device Regulators Forum
MAUDE: Manufacturer and User Facility Device Experience
MHRA: Medicines and Healthcare products Regulatory Agency
ML: machine learning
PRISMA: Preferred Reporting Items for Systematic Review and Meta-Analysis
PRISMA-P: Preferred Reporting Items for Systematic Review and Meta-Analysis Protocols
TGA: Therapeutic Goods Administration
